# The Assessment Rationale of Postgraduate Medical Trainees With Incongruent Self and Faculty Assigned Entrustment Scores

**DOI:** 10.7759/cureus.16666

**Published:** 2021-07-27

**Authors:** Maryam Zadeh, Heather Braund, Timothy Chaplin

**Affiliations:** 1 School of Medicine, Faculty of Health Sciences, Queen's University, Kingston, CAN; 2 Office of Professional Development and Educational Scholarship, Faculty of Health Sciences, Queen's University, Kingston, CAN; 3 Department of Emergency Medicine, Faculty of Health Sciences, Queen's University, Kingston, CAN

**Keywords:** simulation in medical education, medical education assessment, medical education curriculum, medical education and training, medical education research, simulation education, self assessment

## Abstract

Background

Self-assessment is a central skill in competency-based medical education (CBME) and should be fostered in order to promote life-long learning. One measure that will guide the development of self-assessment is the alignment between it and external expert assessment. In this study, we explored the qualitative themes in the self-assessment rationale among trainees with incongruent self and faculty-assigned entrustment scores.

Methods

A total of 40 postgraduate medical trainees completed a four-scenario summative objective structured clinical examination (OSCE) as part of a simulation-based resuscitation curriculum in December 2017. After each scenario, an assessment involving an entrustment score and narrative rationale was completed by both trainee (self) and faculty. The differences between the trainee and faculty scores were calculated for each scenario and summed to give a single “incongruence score”. Trainees who consistently scored themselves higher than the faculty were said to have a “positive-incongruence score” and those scoring below the faculty were said to have a “negative-incongruence” score. Through this method, 10 trainees with the highest and lowest scores were assigned to each group and their narrative rationales were coded and thematically analyzed.

Results

The content of the self-assessment narrative rationale differed between the two groups. Trainees in the positive-incongruence group focused on the concepts of speed and situational management, while trainees in the negative-incongruence group commented on lack of support, and a need to improve communication, diagnosis, and code blue management. The quality of the self-assessment rationale also differed between groups. Trainees in the negative-incongruence group provided higher-quality comments that were more detailed and granular.

Conclusion

We found differences in the content and quality of the self-assessment rationale between trainees whose self and faculty-assigned assessment is incongruent. This provides insight into how these groups differ and has valuable implications for the development of curricula targeting self-assessment skills.

## Introduction

Assessment is a pillar within the competency-based medical education (CBME) paradigm. It serves several purposes, such as providing motivation for learners, direction for future learning, accountability to stakeholders, and support for progression decisions [1]. Importantly, within a CBME framework, assessment drives the learning process through frequent formative feedback based on the direct observation of learners’ abilities.

The traditional construct of assessment in medical education involves an external assessor, usually a faculty member, providing information to a learner. However, self-assessment is recognized as a key metacognitive skill that has implications on patient care and must be developed to maximize life-long learning [2, 3]. Self-assessment is complex and influenced by several factors such as self-confidence, self-efficacy, competence, attitudes, and context-specific experiences [4, 5]. Ideally, a learner’s self-assessment and expert faculty’s assessment would align. However, the literature suggests that a lack of congruence exists and that medical learner’s self-assessment abilities are generally poor [2, 6]. Prior work has demonstrated this incongruence but has not explored how learners with different levels of assessment incongruence approach self-assessment. The qualitative assessment rationale of learners whose self-assessment is higher than faculty assessment compared to those whose self-assessment is lower, may provide insight into the reasons behind this incongruence and help educators develop targeted pedagogical interventions [7].

In this study, we sought to explore the themes within the self-assessment rationale among postgraduate medical trainees whose self-assessment scores were incongruent (both positively and negatively) with faculty-assigned scores in the context of a simulation-based summative objective structured clinical examination (OSCE). 

This article was previously presented as a poster at the following conferences: Queen's Medical Student Research Showcase on October 20, 2020; Ottawa Student Emergency Medicine Conference (OSEM) on January 16, 2021; Ontario Student Medical Education Research Conference (OSMERC) on March 26, 2021; The Canadian Conference on Medical Education (CCME) on April 17-20, 2021; International Conference on Emergency Medicine (ICEM) on June 12, 2021. 

## Materials and methods

We conducted a qualitative study at Queen’s University, an academic centre in Canada. This study is a secondary analysis of the data collected for a larger research study, which examined the feasibility of the Nightmares course and its implication in assessing competence in postgraduate residents’ training [8]. This study was approved by the Health Sciences Research Ethics Board at Queen’s University (REB #: 6028836), and all participants provided informed written consent. 

Participants

Postgraduate medical trainees at the foundations of discipline level of training who participated in the Nightmares Course OSCE during the 2017-2018 academic year were invited to participate in this study. This stage corresponds to the level of trainees in their first postgraduate year, where a breadth of foundational abilities is required before advancing to more specialized training. A total of 53 trainees were invited to participate in the course, 41 of which participated in the OSCE and 40 who gave consent to be included in the study. The Nightmares course at Queen’s University is a longitudinal simulation-based curriculum that aims to teach and assess basic resuscitation skills. Trainees participate in four sessions during the formative portion of the course between August and November, and then a summative OSCE takes place in December. Additional course details can be found in a previous publication about the Nightmares course [8]. 

OSCE

A summative, multi-station simulation-based OSCE occurred in December 2017. The OSCE included four simulation scenarios (opioid overdose, hyperkalemia, septic shock, and myocardial infarction). Each scenario lasted seven minutes with three minutes in between to allow learners to complete a self-assessment and transition to the next room. Trainees participated individually as the leader and one or two registered nurses acted as confederates. Immediately following each scenario, trainees completed a self-assessment that included an entrustment score (Appendix A) and then provided an audio-recorded assessment rationale. A faculty member directly observed each scenario and performed an assessment that included the same entrustment score and narrative rationale (Appendix B). Both assessor groups (faculty and trainee) were prompted to narrate what they thought they did well and what needed further improvement. One faculty assessed each OSCE scenario. All faculty members were comfortable with entrustment scoring and used it regularly in their practice. An orientation was provided on the morning of the OSCE on how to use the assessment tool.

Group assignments

**Figure 1:**
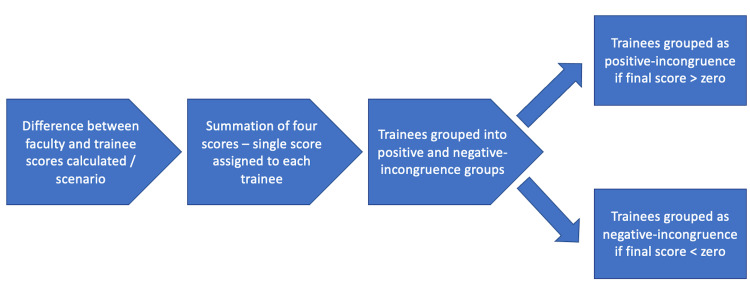
Assigning postgraduate medical trainees into positive-incongruence and negative-incongruence groups

In order to capture those trainees with the most incongruent self and faculty-assigned scores, we calculated the difference between the faculty and trainee entrustment scores for each scenario. These differences were summed, resulting in a single “incongruence score” for each trainee. Those who assigned themselves a higher score than the faculty had a total score above zero and were said to have “positive-incongruence”, and those scoring themselves lower than the faculty had a total score that was negative, and were said to have “negative-incongruence”. Through this method, 10 trainees with the highest scores were assigned to the positive-incongruence group, and 10 with the lowest scores to the negative-incongruence group. Scores were used only to assign groups and were not considered for further analysis. We did not include trainees whose self-assigned scores were more congruent with faculty-assigned scores (i.e., the middle group) as this was not our population of interest. No additional inclusion and exclusion criteria were used other than what is explained above. We included 10 learners in each group as our expected sample size was 40 learners, and groups of 10 would capture the high and low quartiles. An overview of this procedure is represented in Figure 1.

Coding the narrative comments

Narrative rationales for both groups were thematically coded using NVivo software, version 12 (QSR International, Melbourne, Australia). Initially, three trainees from each group were independently coded by two researchers (M.Z. and H.B.), and the results were simultaneously compared for intercoder reliability in order to assure transparency and facilitate communicability and rigour of the coding process [9]. The frequency with which both researchers agreed for each line of coding was totaled and divided according to a total frequency. Thus, an overall percentage of 93% was reached demonstrating reliable and valid coding of the data by the researchers. For the remaining 7% of instances where the two researchers disagreed, they discussed their interpretations until consensus (100% agreement) was reached. This process resulted in a consensus-built codebook that was used by one researcher for the remainder of the coding process. Subsequently, the narrative comments for the rest of the trainees were independently coded in order to identify emerging themes that arose between the two groups. Data saturation was reached after coding data from eight participants within each group. To verify, the researcher coded two additional participants in each group, but no new patterns emerged. The researcher (M.Z.) assigned codes line by line, grouped similar codes together into subthemes, and finally similar subthemes into overall themes. The researchers met throughout the coding process to ensure agreement and a common understanding of the coded data. Once preliminary themes were identified, the whole research team met to discuss data interpretation and organization of themes and subthemes. 

Emergent finding: quality of self-assessment

During the process of qualitative analysis, two authors (M.Z. and H.B.) identified another emergent theme that was inherently different between the two groups. The coders noticed that there were better descriptors and longer answers provided by learners in the negative-incongruence group. In order to analyze this, the researchers coded the qualitative data based on whether the trainees made “high quality” or “low quality” comments, using an adapted version of the framework described by Abraham and Singaram (2019) [10]. More specifically, we used their qualitative descriptors to identify what was considered a high-quality versus a low-quality comment. The comments were coded as “high quality” if they were specific and detailed. In other words, if the content, value, specific skills, and tasks were described in the self-assessment, they were coded as “high quality”. For example, a comment such as “I believe my initial management was adequate with vital signs, IV, and oxygen” was coded as high quality. The self-assessment was coded as “low quality” if they were vague, general comments without indication of any specific behavior, tasks, or learning goals. For example, a comment such as “I think I managed the situation well” was coded as a low-quality comment. 

Research team and reflexivity 

The primary investigator (T.C.) was the course director and one of the course instructors. He developed and managed the OSCE. He is a full-time clinician-educator in emergency medicine at Queen’s University. He directly worked with the study participants during the formative portion of the course. H.B. distributed and collected the post-OSCE self-assessment forms. She is a PhD-trained mixed methods researcher in health sciences and education. M.Z. conducted the data analysis. She is a medical student at Queen’s University, with a Master of Science degree and background in scientific research. The data analysis was conducted under the supervision of H.B. M.Z. was the primary researcher who assigned residents into positive- and negative-incongruence groups before coding. However, H.B. was blinded to the groupings, and M.Z. approached the coding across groups. These efforts were put in place to help reduce potential bias from the knowledge of trainees’ groupings. Lastly, M.Z. did not have any direct contact or experience working with the study participants, helping to mitigate potential biases. 

## Results

Participants

All 40 first-year postgraduate medical trainees and eight faculty who participated in the Nightmares Course OSCE provided consent and completed an assessment following each scenario. 10 trainees were assigned to both the positive-incongruence and negative-incongruence groups. The positive-incongruence group consisted of eight males (80%) and two females (20%), and the negative-incongruence group consisted of six males (60%) and four females (40%). The average age range for both groups was 26-30 years old. The participants represented 12 different training programs, including anesthesiology, general surgery, internal medicine, neurology, obstetrics and gynecology, ophthalmology, orthopedic surgery, pathology, physiatry, psychiatry, diagnostic radiology, and urology.

Self-assessment rationale

**Figure 2:**
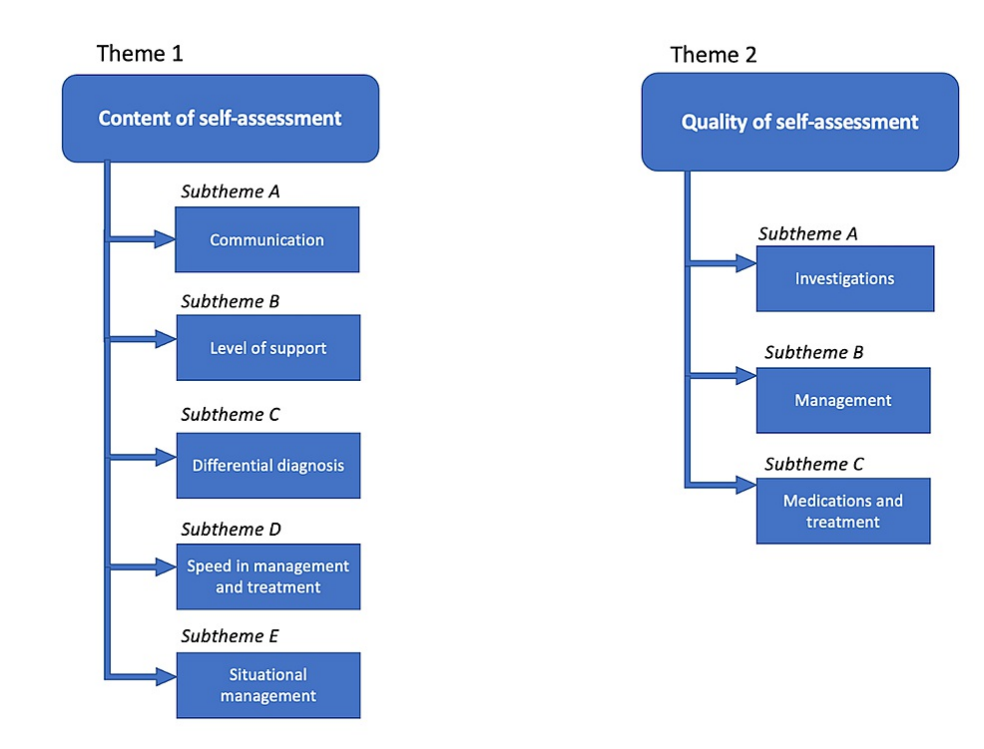
Overview of themes and subthemes from qualitative data

Two broad themes emerged from the self-assessment rationale that differed between the positive-incongruence and negative-incongruence groups. These included both the content and the quality of the self-assessment rationale. Within each theme, subthemes emerged that substantiate the differences between the groups (Figure 2). 

Content of self-assessment

A. Communication 

Comments related to communication were approached differently between trainees in the two groups. The negative-incongruence group focused on communication as an area requiring further improvement, with four of the trainees commenting on a need to communicate more effectively with the team and the patients. Only one of the 10 trainees in the positive-incongruence group mentioned communication as an area requiring improvement. 

B. Level of Support

The perceived level of support also differed in the narrative rationale between the two groups. Six trainees in the negative-incongruence group noted that they required support from other team members and felt that there was a lack of appropriate help during the scenario. Only two trainees in the positive-incongruence group highlighted this subtheme in their self-assessment rationale.

C. Differential Diagnosis

The subtheme of establishing a differential diagnosis differed between the two groups. Four trainees in the negative-incongruence group commented on keeping a broad differential, awareness of having an adequate diagnosis or refraining from having a premature diagnosis. Only one trainee in the positive-incongruence group commented on this subtheme. 

D. Speed in Management and Treatment 

Trainees in the positive-incongruence group made frequent comments related to speed compared to the negative-incongruence group. For example, they highlighted speed in administering treatment as an area requiring improvement. Three of the trainees in the positive-incongruence group noted that they should have gone through the treatment more rapidly and administered it more quickly, whereas the concept of speed was not mentioned in the negative-incongruence trainees’ self-assessment. Interestingly, the trainees in the positive-incongruence group also highlighted the concept of speed in relation to clinical assessment. Six of the trainees commented on rapidly assessing the situation, quickly recognizing the problem, and acting upon the issue as an area of strength. Only two trainees in the negative-incongruence group highlighted these areas.

E. Situational Management 

Trainees in the positive-incongruence group commented on broad aspects of situational management as an area requiring improvement. For example, four trainees commented on notions such as the need to navigate the scenario when no support is present and manage the scenario with multiple people present. None of the trainees in the negative-incongruence group made any statements about broad situational management; however, three of them specifically highlighted code blue management as an area requiring further improvement.

Example quotations from the theme of content are presented in Table 1. 

**Table 1:** Sample quotations that differed between the positive-incongruence and negative-incongruence groups within the theme of content

Subtheme	Quotations
A. Communication	"In terms of the improvement, I think I should call my senior earlier and communicate.” (Negative-incongruence P33) , “Increased communication would be a primary working point for this scenario.” (Negative-incongruence P6)
B. Level of Support	"There was a limited help, I don’t know if that was part of the scenario?” (Negative-incongruence P2) , "It would’ve been helpful to have cardiology call back." (Negative-incongruence P25)
C. Differential Diagnosis	“What could be improved? Man, I don’t have a diagnosis.” (Negative-incongruence P27) , “What I feel I could’ve done better is I could’ve done a bit more of a search of alternate causes.” (Negative-incongruence P40)
D. Speed in Management and Treatment	“Going through treatments a little quicker, I guess, with mona, morphine, oxygen, nitro, and aspirin." (Positive-incongruence P14) , "Things I did really well, is I think I recognized pretty immediately this was a case of opioid toxicity." (Positive-incongruence P16)
E. Situational Management	“I think navigating that situation and reflecting on that, maybe going forward of what to do when no one shows up.” (Positive-incongruence P26) , "...a little less confident with running a code which I’ve never done before so that’s something needs to be worked on." (Negative-incongruence P4)

Quality of self-assessment 

The quality of narrative self-assessment differed between the trainees in the positive-incongruence and negative-incongruence groups; specifically, the granularity and level of detail. As a group, the trainees in the positive-incongruence group made lower quality comments compared to the negative-incongruence group within topics such as investigations, management, medication, and treatment. 

A. Investigations

The two groups differed in the quality of their narrative assessment rationale related to investigations. Five trainees in the negative-incongruence group made comments about investigations that were of high quality, compared with three in the positive-incongruence group. Only one trainee in the negative-incongruence group made a low-quality comment within this subtheme compared to seven in the positive-incongruence group.

B. Management

Eight trainees in the negative-incongruence group made high-quality comments about management, compared to two trainees in the positive-incongruence category. Conversely, six trainees in the positive-incongruence group made general and vague comments about management, compared to only two trainees in the negative-incongruence category. 

C. Medication and Treatment 

Almost every participating trainee made some remark about medication and treatment. However, the quality of the comments differed between the two groups. Eight trainees in the negative-incongruence group made high-quality comments surrounding areas of medication and treatment, compared to five trainees in the positive-incongruence category. On the other hand, four trainees in the positive-incongruence group made low-quality comments, compared to one trainee in the negative-incongruence group. 

Example quotations from the theme of quality are presented in Table 2. 

**Table 2:** Sample quotations from positive-incongruence and negative-incongruence trainees’ self-assessment, comparing the quality of feedback made about different subthemes

Subtheme	Quality	Quotations
A. Investigations	High quality	“Vital signs were stable, and after looking at the monitor and discussing with the nurse, discovered that he was accidentally given too much oral narcotic.” (Negative-incongruence P1)
A. Investigations	Low quality	“I think I started all the appropriate investigation” (Positive-incongruence P22)
B. Management	High quality	“I still need to work on making a call to initiate the appropriate management. For example, probably needed to get anticoagulation on board before cardiology got there as he was symptomatically unstable.” (Negative-incongruence P27)
B. Management	Low quality	“I prioritized management, over you know, wondering why this was happening.” (Positive-incongruence P16)
C. Medications and Treatment	High quality	“The patient was unresponsive, she had error of giving 5 mL of Dilaudid. I immediately gave her Narcan.” (Negative-incongruence P33)
C. Medications and Treatment	Low quality	“I think maybe I could’ve [got] my treatment in a little faster.” (Positive-incongruence P16)

## Discussion

We found differences in the content and quality of narrative self-assessment rationale of trainees whose self-assessment scores were incongruent (either positively or negatively) with faculty-assigned scores in the context of a summative simulation-based resuscitation OSCE. Within the themes of content and quality, subthemes emerged that substantiate the differences between the two groups. Trainees in the negative-incongruence group focused on improving communication skills, establishing a differential diagnosis, and code blue management. As a group, they also provided higher-quality feedback compared to the positive-incongruence group. Trainees in the positive-incongruence group focused on broad concepts of situational management and speed in their assessment and management of the scenario. These findings provide insight into how learners approach self-assessment and have implications for future curricula targeting the development of self-assessment skills.

Self-assessment is a skill that requires attention within a CBME curriculum [11]. Previous research indicates that the capacity for self-assessment amongst medical learners and physicians at all levels is generally poor [4]. The process of self-assessment is not intuitive and learners are often told to perform this activity with little to no guidance [12, 13]. Understanding content and quality differences in the self-assessment rationale of trainees with poor congruence between self-assigned performance scores and the faculty’s rating is significant as it can help us to conceptualize the components and processes that comprise self-assessment and the factors that influence them. 

It may be assumed that learners in the high-incongruence group, i.e., those who consistently scored themselves higher than the faculty, did so because they were overconfident. Self-confidence is the perception and measure of one’s skills and abilities and is important within the context of performance and skill acquisition [14]. While there may be a readily accepted view that high levels of self-confidence can enhance performance, a growing body of literature has reported otherwise [15, 16]. For example, Vancouver and colleagues (2001) found that overconfidence was associated with poor performance, as learners may not feel that they need to invest effort in the tasks [16]. Bandura and Locke (2003) suggest that some self-doubt in one’s performance is necessary to provide an incentive to acquire knowledge and master a skill [17]. This relationship likely translates to medical trainees, and some degree of self-doubt may provide an incentive to consider and understand a knowledge gap and then obtain the required skills for improvement. During this process, self-assessment and reflection should both accurately identify one’s strengths, and more importantly, address gaps in skills and knowledge [4]. Our finding that the self-assessment rationale of the trainees in the positive-incongruence group lacked granularity and detail suggests that overconfidence may be associated with poor-quality self-reflection.

Educators are often encouraged to identify factors that increase skill acquisition and knowledge retention in order to encourage self-regulated and life-long learning [18, 19]. Identifying differences in the narrative rationale between trainees in the two groups can provide faculty with areas of focus when providing feedback and can facilitate the development and design of programs to aid skill development and self-regulated learning [20]. For example, within a simulation context, if a trainee is identified as consistently being “positively-incongruent”, educators might focus time and attention on the details of the scenario and encourage critical reflection. Throughout training and continuing as practicing physicians, insight into performance and the ability to conduct accurate and constructive self-assessment should be fostered [21]. Many postgraduate learners enter residency dependent on external sources such as defined curricula and faculty-guided assessment and feedback. Similar to other skills that must be acquired during training, the process of self-assessment should be an explicit competency that begins as a shared responsibility between faculty and trainee in the early stages, but then shifts to become an independent process as the trainee gains proficiency in self-assessment [12]. Knowing the focus of trainee self-assessment can guide educators to help trainees calibrate and align their self-assessment to be constructive and facilitate life-long self-regulated learning. Future research is required to define the most effective and efficient approach to develop the metacognitive skills of learners, as well as the link between self-assessment and life-long learning outcomes.

Our study has several limitations. Firstly, our results may not be generalizable to other contexts or learner groups as this was a single-centre study involving postgraduate medical trainees. Secondly, the setting was a simulation-based OSCE and trainees were primed to provide feedback on their performance. This may not be generalizable to performance and self-reflection in the “real world”. Lastly, one of the study’s researchers primarily grouped trainees into their respective categories prior to coding and this may have introduced bias. The second researcher was blinded to the grouping, which may have reduced potential bias. 

## Conclusions

The self-assessment rationale differed in content and quality between trainees whose self-assessment scores were incongruent (either positively or negatively) with faculty-assigned scores in the context of a summative simulation-based resuscitation OSCE. These findings suggest that learners with varying levels of assessment incongruence approach do self-assessment with different priorities and self-reflection abilities. Future efforts are required to support CBME curricula targeting the development of self-assessment skills.

## Figures and Tables

**Figure 3:**
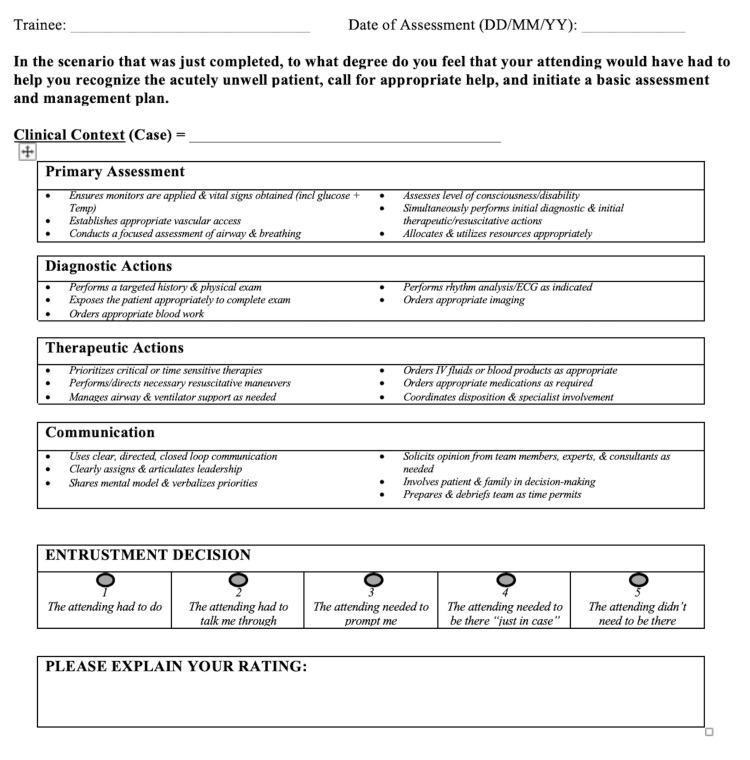
Trainee Nightmares Course and OSCE Assessment Form (Appendix A)

**Figure 4:**
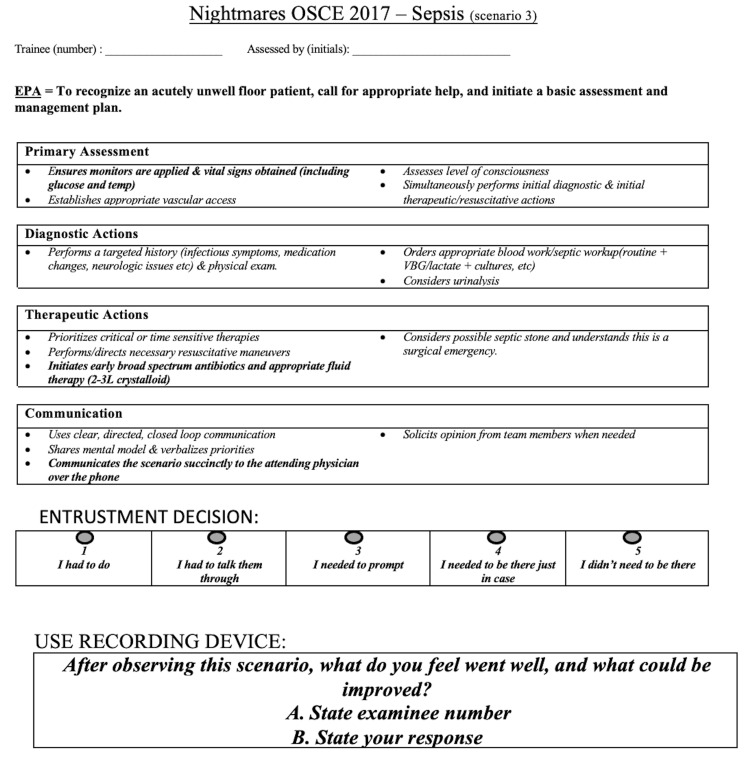
Evaluator Nightmares Course and OSCE Assessment Form (Appendix B)

## References

[1] Epstein RM (2007). Assessment in medical education. N Engl J Med.

[2] Davis DA, Mazmanian PE, Fordis M, Van Harrison R, Thorpe KE, Perrier L (2006). Accuracy of physician self-assessment compared with observed measures of competence: a systematic review. JAMA.

[3] Westberg J, Jason H (1994). Fostering learners' reflection and self-assessment. Fam Med.

[4] Eva KW, Regehr G (2005). Self-assessment in the health professions: a reformulation and research agenda. Acad Med.

[5] Mavis B (2001). Self-efficacy and OSCE performance among second year medical students. Adv Health Sci Educ Theory Pract.

[6] Schneider JR, Verta MJ Jr, Ryan ER, Corcoran JF, DaRosa DA (2008). Patient assessment and management examination: lack of correlation between faculty assessment and resident self-assessment. Am J Surg.

[7] Mattheos N, Nattestad A, Falk-Nilsson E, Attström R (2004). The interactive examination: assessing students' self-assessment ability. Med Educ.

[8] McMurray L, Hall AK, Rich J, Merchant S, Chaplin T (2017). The Nightmares course: a longitudinal, multidisciplinary, simulation-based curriculum to train and assess resident competence in resuscitation. J Grad Med Educ.

[9] O’Connor C, Joffe H (2020). Intercoder reliability in qualitative research: debates and practical guidelines. Int J Qual Methods.

[10] Abraham RM, Singaram VS (2019). Using deliberate practice framework to assess the quality of feedback in undergraduate clinical skills training. BMC Med Educ.

[11] Ramani S, Krackov SK (2012). Twelve tips for giving feedback effectively in the clinical environment. Med Teach.

[12] Keister DM, Hansen SE, Dostal J (2017). Teaching resident self-assessment through triangulation of faculty and patient feedback. Teach Learn Med.

[13] Sargeant J, Armson H, Chesluk B (2010). The processes and dimensions of informed self-assessment: a conceptual model. Acad Med.

[14] Perry P (2011). Concept analysis: confidence/self-confidence. Nurs Forum.

[15] Vancouver JB, Thompson CM, Tischner EC, Putka DJ (2002). Two studies examining the negative effect of self-efficacy on performance. J Appl Psychol.

[16] Vancouver JB, Thompson CM, Williams AA (2001). The changing signs in the relationships among self-efficacy, personal goals, and performance. J Appl Psychol.

[17] Bandura A, Locke EA (2003). Negative self-efficacy and goal effects revisited. J Appl Psychol.

[18] Andrade HL (2019). A critical review of research on student self-assessment. Front Educ.

[19] Clanton J, Gardner A, Cheung M, Mellert L, Evancho-Chapman M, George RL (2014). The relationship between confidence and competence in the development of surgical skills. J Surg Educ.

[20] Egan R, Chaplin T, Szulewski A (2020). A case for feedback and monitoring assessment in competency-based medical education. J Eval Clin Pract.

[21] Wolff M, Santen SA, Hopson LR, Hemphill RR, Farrell SE (2017). What's the evidence: self-assessment implications for life-long learning in emergency medicine. J Emerg Med.

